# Abnormal sensory sensitivity during pregnancy, peri-partum and post-partum in mothers with high functioning autism spectrum disorder: A preliminary study

**DOI:** 10.1192/j.eurpsy.2021.1605

**Published:** 2021-08-13

**Authors:** V. Nisticò, S. Limonta, R. Faggioli, P. Turrizziani, L. Gianbanco, F. Calistro, A. Priori, B. Demartini, O. Gambini

**Affiliations:** 1 Dipartimento Di Scienze Della Salute, Università degli Studi di Milano, Milano, Italy; 2 “aldo Ravelli” Research Center For Neurotechnology And Experimental Brain Therapeutics, Università degli Studi di Milano, Milano, Italy; 3 Unità Di Psichiatria Ii, ASST Santi Paolo e Carlo, Presidio San Paolo, Milano, Italy; 4 Dipartimento Di Scienze Psicologiche, Pedagogiche, Dell’esercizio Fisico E Della Formazione, Università degli Studi di Palermo, Palermo, Italy; 5 Unità Di Ostetricia E Ginecologia, Ospedale S Antonio Abate, Erice, Italy; 6 Iii Clinica Neurologica, ASST Santi Paolo e Carlo, Presidio San Paolo, Milano, Italy; 7 Unità Di Psichiatria Ii, ASST Santi Paolo e Carlo, Presidio San Paolo, Milano, Italy

**Keywords:** Sensory perception, pregnancy, post-partum, High Functioning Autism Spectrum Disorder

## Abstract

**Introduction:**

Individuals with Autism Spectrum Disorder without intellectual disabilities (High Functioning ASD, HF-ASD) present atypical sensory sensitivity, due to the hyper-reactivity to sensory inputs.

**Objectives:**

To retrospectively evaluate the sensory sensitivity in a sample of mothers with HF-ASD during pregnancy (pre-partum), delivery and childbirth (peri-partum) and during the three months after delivery (post-partum).

**Methods:**

19 HF-ASD and 13 neurotypical (NT) mothers were asked to complete an ad-hoc questionnaire designed for the study, named Maternity Questionnaire, assessing sensory perception during pre-, peri- and post-partum. Moreover, they underwent the following assessment: the Autism-Spectrum Quotient (AQ), the Empathy Quotient (EQ), the Ritvo Autism Asperger Diagnostic Scale-Revised (RAADS-R), the Edinburg Postnatal Depression Scale (EPSD), the Sensory Perception Quotient (SPQ) and the Post Partum Bonding Questionnaire (PBQ).

**Results:**

At the Maternity Questionnaire, HF-ASD mothers showed higher sensitivity scores than NT mothers overall. Moreover, HF-ASD mothers presented lower sensitivity during the peri-partum, compared to pre- and post- partum periods, while NT mothers showed a linear decrease from pre- to post- partum. The two groups significantly differed at the AQ, the EQ, the RAADS-R, the SPQ and Factor 3 of the PBQ. Sensitivity during pre- and post-partum positively correlated with EPDS scores.

**Conclusions:**

Mothers with HFA tend to experience pregnancy, childbirth and the post-partum period differently from neurotypical mothers, particularly in terms of hypersensitivity, although during the peri-partum the hypersensitivity decreases. Further studies investigating these aspects might give fundamental hints to provide proper help to HF-ASD mothers during pregnancy and motherhood overall.

